# Effect of regulatory cell death on the occurrence and development of head and neck squamous cell carcinoma

**DOI:** 10.1186/s40364-022-00433-w

**Published:** 2023-01-05

**Authors:** Yuting Xue, Xuejiao Jiang, Junrong Wang, Yuxuan Zong, Zhennan Yuan, Susheng Miao, Xionghui Mao

**Affiliations:** 1 Department of Head and Neck Surgery, Harbin Medical University Cancer Hospital, Harbin, China; 2 Beijing Key Lab of TCM Collateral Disease Theory Research, School of Traditional Chinese Medicine, Capital Medical University, Beijing, China; 3 Department of Breast Surgery, The First of hospital of Qiqihar, Qiqihar, China

## Abstract

Head and neck cancer is a malignant tumour with a high mortality rate characterized by late diagnosis, high recurrence and metastasis rates, and poor prognosis. Head and neck squamous cell carcinoma (HNSCC) is the most common type of head and neck cancer. Various factors are involved in the occurrence and development of HNSCC, including external inflammatory stimuli and oncogenic viral infections. In recent years, studies on the regulation of cell death have provided new insights into the biology and therapeutic response of HNSCC, such as apoptosis, necroptosis, pyroptosis, autophagy, ferroptosis, and recently the newly discovered cuproptosis. We explored how various cell deaths act as a unique defence mechanism against cancer emergence and how they can be exploited to inhibit tumorigenesis and progression, thus introducing regulatory cell death (RCD) as a novel strategy for tumour therapy. In contrast to accidental cell death, RCD is controlled by specific signal transduction pathways, including TP53 signalling, KRAS signalling, NOTCH signalling, hypoxia signalling, and metabolic reprogramming. In this review, we describe the molecular mechanisms of nonapoptotic RCD and its relationship to HNSCC and discuss the crosstalk between relevant signalling pathways in HNSCC cells. We also highlight novel approaches to tumour elimination through RCD.

## Introduction

Head and neck squamous cell carcinoma (HNSCC) is one of the most common malignancies worldwide, with an annual incidence of more than 780,000 cases. In the past few decades, the treatment of HNSCC, including surgery, radiotherapy, and chemotherapy, has made significant progress, but the mortality rate is still as high as 40–50% [1–3]. HNSCC is highly invasive and involves lesions in the oral cavity, oropharynx, larynx, and hypopharynx. Common risk factors are smoking, excessive alcohol consumption, and human papillomavirus (HPV) infection [4, 5]. Because the early symptoms of HNSCC are not obvious or specific, patients are often in an advanced state at the initial stage of diagnosis [6]. Therefore, it is necessary to gain a more systematic understanding of the molecular mechanisms and signalling pathways of cell death in HNSCC and to explore new treatment regimens to prolong the survival rate of patients.

**Fig. 1:**
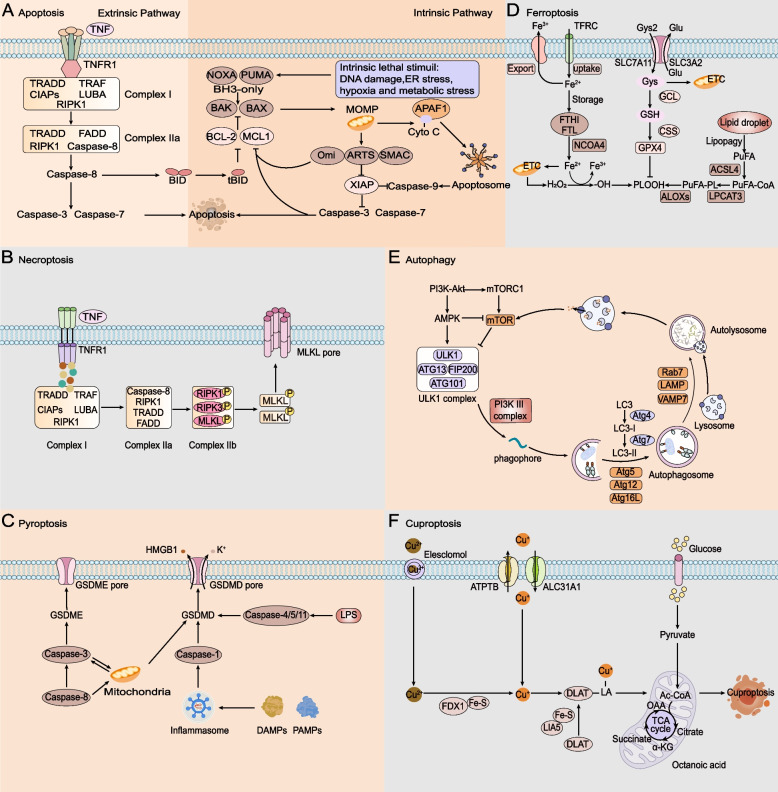
Core molecular mechanisms of apoptosis, necroptosis, pyroptosis, ferroptosis, autophagy and cuproptosis. **A** In the exogenous apoptosis pathway, TNF interacts with TNFR1, and TNFR1 begins to recruit downstream protein molecules to form complexes I and IIa, which promote the activation of caspase-8 and then activate caspase-3 and caspase-7. In the endogenous apoptotic pathway, activation of BH3-only proteins leads to Bax and BAK activity, triggering MOMP. Cyto C is released from mitochondria and forms a multiapoptotic complex with APAF1, which activates caspase-9 and then caspase-3 and caspase-7, leading to apoptosis. **B** TNF interacts with TNFR1; when caspase 8 is inhibited, activated RIPK1 promotes RIPK3 recruitment and MLKL phosphorylation, forms complex IIb, promotes inflammatory signal secretion, and promotes necroptosis. **C** PAMP and DAMP stimulate inflammasome activation, which leads to caspase-1 cleavage, and LPS can bind to caspase-4/5/11 to lyse GSDMD. Potassium efflux triggers the release of HMGB1 and K+. Caspase-3 can also be activated through the mitochondrial endogenous pathway and death receptor pathway to lyse GSDME and trigger pyroptosis. **D** Iron accumulation is achieved by increasing iron uptake by the TF-TFRC complex, limiting iron efflux by iron export transporters, and reducing iron storage by ferritosis. Cells obtain cysteine and exchange it for glutamate through the XC − antitransporter system. The ACSL4-LPCAT3-AlOXs pathway promotes iron death by activating lipid peroxidation to produce PLOOH from polyunsaturated fatty acids. **E** AMPK and mTORC1 act on mTOR, the ULK1 complex is phosphorylated, and the PI3K complex interacts with autophagosomes. Lc3 is modified to form LC3-II and ATG5-ATG12-ATG16L complexes to promote the formation and maturation of autophagic vesicles and binds to lysosomes under the action of LAMP, Rab7 and VAMP7. The recovered product is released into the cytosol for reuse. **F** Elesclomol binds extracellular copper (Cu2+) and transports it into the cell. FDX1 reduces Cu2 + to Cu + and promotes lipolylation (LA) and aggregation of DLAT involved in the mitochondrial TCA cycle. Copper importers (e.g., SLC31A1) and exporters (e.g., ATP7B) modulate copper sensitivity by affecting intracellular copper ion levels

Cell death is the irreversible cessation of life phenomenon and the end of life. Cell death often occurs in normal tissues and is necessary to maintain tissue function and morphology [7]. On the one hand, cell death is an essential physiological process that maintains tissue homeostasis and ensures normal development of the body. On the other hand, it is also the primary pathogenic mechanism that leads to local or systemic inflammation and disrupts normal organ function [8]. The modes of cell death can be divided into two main categories: accidental cell death (ACD) and regulatory cell death (RCD) [9]. The former is a passive biological process not regulated by life activities. At the same time, the latter occurs as a programmed regulation characterized by controlled signalling pathways and precise effector mechanisms [10]. RCD can be subdivided into apoptosis, necroptosis, pyroptosis, ferroptosis, autophagy, and the recently discovered cuproptosis (Table 1; Fig. 1). Tumours are diseases characterized by an imbalance in cell proliferation, differentiation, and cell death. Selective elimination of cancer cells without damaging nonmalignant cells is an ideal treatment strategy for most cancers [11] Death resistance is an essential characteristic of tumour cells, and inducing cell death is a necessary strategy for the nonsurgical treatment of tumours [12]. Different lethal substances in the RCD process affect tumour development and therapeutic effects. RCD processes involve signalling cascades of effector molecules and have unique biochemical characteristics, morphological characteristics, and immunological consequences. Apoptosis, necroptosis, pyroptosis, autophagy, ferroptosis, and cuproptosis have been the most widely studied RCDs in recent years, and different RCDs have unique molecular mechanisms [13].

**Table 1:** Morphological, biochemical,immune features and major regulators of apoptosis,necroptosis,pyroptosis,ferroptosis,autophagy and cuproptosis

Type	Morphological features	Biochemical features	Immune features	Core regulators
Apoptosis	Cell shrinkage,chromatin condensation, cell fragmentation, apoptotic body formation	Activation of caspases,DNA fragmentation, formation of apoptosome	TCD or ICD	Positive:AIF,APAF1,BAX,BAK1,BBC3(PUMA),BID,PMAIP1(NOXA) and TP53 Negative: BCL-2, BCL-XL and MCL1
Necroptosis	Cells swelling, pore formation on cells membranes,plasma membranes rupture, and moderate chromatin condensation	Caspase-independent,RIPK1/RIPK3-mediated phosphorylation of MLKL, formation of necrosome	ICD	Positive: MLKL, RIPK1 and RIPK3 Negative:AURKA and ESCRT-III
Pyroptosis	Cell membrane perforation, cellswelling,osmotic lysis, release of contents	Caspase-dependent ,GSDMD or GSDME cleavage,formation of inflammasome	ICD	Positive:Caspase-1,Caspase-4, Caspase-5,Caspase-11,GSDMD and GSDME Negative: ESCRT-III and GPX4
Ferroptosis	Cell swelling, pore formation on cell membrane, smaller mitochondria, reduced mitochondria crista, elevated mitochondrial membrane densities	Caspase-independent, iron accumulation, lipid peroxidation, damage to the antioxidant system, inhibition of system Xc–/GSH/GPX4 signalling	ICD	Positive:ACC,ACSL4,ALOX, CDKN1A,DPP4,LPCAT3, NCOA4 and TFRC Negative:GPX4, HIF1α and SLC7A11
Autophagy	Phagosomes, autophagosomes, autolysosomes	Caspase-independent, LC3 lipidation, formation of autophagosome, elevated autophagic flux, and lysosomal activity	ICD	Positive: AMPK,ULK1 and PI3K Negative: mTOR
Cuproptosis		Caspase-independent,copper accumulation,aggregation of lipoacylated proteins	ICD	Positive:FDX1,LIAS,LIPT1,DLD,DLAT,PDHA1 and PDHB Negative:MTF,GLS and CDKN2A

## Mechanisms of cell death in HNSCC

### Apoptosis

Apoptosis is a cell death induced by cysteine aspartate aminotransferase (caspase) bound by the mitochondrial outer membrane permeability (MOMP) [14]. The morphology is characterized by cell shrinkage, chromatin condensation, and cell fragmentation to form so-called apoptotic bodies, which are phagocytosed by neighbouring or specific cells [15]. Apoptosis occurs mainly through two pathways. One pathway, called the endogenous pathway, is initiated by mitochondria. Cytochrome C release is activated by proapoptotic members, such as the apoptosis regulators BAX, BCL-2 homologous antagonist/killer 1 (BAK1), BCL-2-binding component 3 (BBC3; also known as PUMA), BH3 interacting domain death agonist (BID) and BH3-only proteins, and is stimulated by the apoptosis regulators BCL-2 (BCL-2), BCL-2-like 1 (BCL-2L1; also known as BCL-XL), BCL-2L2 (also known as BCLW) and MCL1 of the BCL-2 protein family to alter the permeability of the mitochondrial outer membrane [16, 17]. Cyto C released from mitochondria binds to the apoptotic peptidase activating factor 1 (APAF1), induces the recruitment, oligomerization, and activation of the apoptotic initiation protein caspase-9 in the conformational complex of the endoplasmic reticulum, and subsequently cleaves and activates the effector proteins caspase-3 and caspase-7 to induce apoptosis. Inhibition of caspase-9 is mediated by X-linked inhibitor of apoptosis (XIAP), which is degraded by the inhibitor of apoptosis (IAP) antagonists SMAC, Omi, and ARTS during cell death [15]. The other pathway is exogenous and is initiated through cell-surface death receptors of the tumour necrosis factor (TNF) family. TNF interacts with tumour necrosis factor receptor 1 (TNFR1), and TNFR1 begins to recruit downstream proteins, such as cytosolic adaptor protein receptor-interacting serine/threonine-protein kinase 1 (RIPK1), cellular IAPs (cIAPs), TNFR­associated factors (TRAFs) and linear ubiquitin chain assembly complex (LUBAC) proteins, to form complex I, which is involved in nuclear factor B (NF-B)-dependent survival gene expression. [18] TRADD, FAS-associated death domain (FADD), caspase-8, and RIPK1 constitute complex IIa and activate the apoptosis-initiating protein caspase-8, which cleaves and activates the effector proteins caspase-3 and caspase-7 to induce apoptosis [15, 19].

MCL-1 and XIAP are antiapoptotic proteins that induce apoptosis through caspase-activated cleavage inactivation [20]. G-protein-coupled receptor kinase-interacting protein 1 (GIT1) is upregulated in HNSCC to inhibit the apoptosis pathway through the PI3K/AKT/mTOR pathway, thereby promoting the development of HNSCC cells. Therefore, we believe that GIT1 may be a carcinogen of HNSCC and promote the occurrence and development of tumour, or may be a potential target for HNSCC therapy by inhibiting GIT1. [21, 22]. NF-κB is a key protein in regulating apoptosis, both to protect cells from apoptosis and to promote apoptosis in HNSCC cells in other cases. By inhibiting the activity of NF-κB in tumour cells, apoptosis procedures can be activated, so modulating the nuclear factor κB pathway may provide a new therapeutic strategy for HNSCC [23, 24]. WEE-1 is a serine/threonine kinase that inactivates cyclin-dependent kinase 1 (CDK1) by phosphorylation of the Tyr15 residue, resulting in G2/M phase arrest. Therefore, AZD-1775, an inhibitor of WeE-1, can overturn G2 cell cycle arrest and cause DNA damage by disrupting genome replication and can then promote the apoptosis of HNSCC [25–27]. The combination of cisplatin and AZD-1775 can produce persistent DNA damage in HPV + HNSCC cancer cells, downregulate MCL-1 and XIAP, and induce apoptosis in HPV + tumour cells [28, 29].

### Necroptosis

For several years, apoptosis has been considered the simplest form of RCD, even as necroptosis seems to occur in an ACD manner. Genetic, biochemical, and functional evidence, as well as the discovery of specific chemical inhibitors of necrosis, redefine this process as a form of molecular-controlled regulation of cell death [30]. In terms of pathological functions, necroptosis does not involve apoptotic bodies; there is no great change in chromatin, the integrity of the cell membrane is destroyed, and cells and organelles are swollen, inflicting an excessive inflammatory response [31, 32]. The common pathway of necroptosis is initiated by activating receptor-interacting protein kinase-1 (RIP1). After the interaction between TNF and TNFR1, TNFR1 begins to recruit the downstream protein molecules TRADD, RIPK1, cIAPs, TRAF, and LUBAC to form complex I. Complex IIa is composed of TRADD, FADD, caspase-8, and RIPK1. When caspase-8 is inhibited, activated RIPK1 promotes RIPK3 recruitment and mixed lineage kinase domain-like (MLKL) phosphorylation, forms complex IIb, promotes proinflammatory signals such as DAMP secretion, and promotes necroptosis [33–35].

Chemotactic cells can promote the migration and invasion of HNSCC cells by means of freeing damage-related molecular patterns (DAMPs) and RIPK1, activating the nuclear factor-κB (NF-κB) pathway in HNSCC, and hence increasing the migration, invasion, and proliferation of tumour cells [36]. The determinant of the necrotic reaction is the RIPK3 receptor interaction. Cells expressing RIPK3 can be necrotic under the action of cytokines of the TNF family [37]. In patients with HNSCC, the loss of RIPK1 and RIPK3 function caused by hypermethylation of the promoter is closely related to metastatic disease and poor prognosis [38, 39]. Under normal circumstances, necroptosis is considered to back apoptosis because caspase-8 is a potent inhibitor of this process. However, some researchers have proven that caspase-8 is one of the genes with the highest mutation frequency in HNSCC, and it is also an essential factor leading to apoptosis resistance in HNSCC cells. Therefore, targeted necroptosis may provide a new strategy for HNSCC to bypass apoptosis resistance and eliminate tumour cells [40]. There is growing evidence that necroptosis plays a crucial role in regulating tumorigenesis and cancer progression, but it seems to be a double-edged sword [41]. Inducing necroptosis of cancer cells is a method for treating cancer, but necroptosis is also involved in the pathogenic process of tumour cells. The linear ubiquitin chain assembly complex (LUBAC) can also mediate NF-κB signalling and induce tumour cell death resistance [42]. Necroptosis can promote extravasation and migration of tumour cells. For example, tumour cells can induce necroptosis of endothelial cells through death receptor 6 (DR6) on endothelial cells, which leads to extravasation and metastasis of tumour cells mediated by the expression of amyloid precursor protein [43].

### Pyroptosis

Pyroptosis is a programmed cell death mediated by cysteine aspartate protease 1 (caspase-1) and is characterized by cell membrane perforation, cell swelling, osmotic lysis, and content release [44, 45]. Unlike apoptosis, pyroptosis induces inflammation by releasing proinflammatory cytokines (IL-1β and IL-18) and cellular contents. Gasdermin (GSD) family proteins are the main executors of pyroptosis and mediate the formation of cell membrane perforation [46, 47]. Depending on the activated cysteine aspartate aminotransferase, typical and atypical signalling pathways may trigger pyroptosis [48]. The classical cellular pyroptosis pathway is mediated by caspase-1. [49] The inflammatory body is a kind of intracellular multiprotein signal complex that is usually assembled around the pattern recognition receptors (PRRs) [50]. Pathogen-associated molecular patterns (PAMPs) and DAMPs bind to adapter proteins on apoptosis-related protein (ASC) to activate caspase-1. Then, GSDMD is cleaved as a cutting substrate, resulting in cellular pyroptosis [51, 52]. The nonclassical pathway relies on caspase-11 (mouse) and caspase-4/5 (human). Pattern recognition receptors recognize the binding of Gram-negative bacterial lipopolysaccharide (LPS) to caspase-4/5/11 to cleave GSDMD, and potassium efflux-regulated pyroptosis triggers the release of high mobility group box 1 (HMGB1) and K+, leading to pyroptosis [43, 53, 54].

Inflammation is one of the causes of HNSCC, and the development and function of tumour stem cells may be related to chronic inflammation [55]. Inflammatory bodies and gasdermin family proteins are critical substrates leading to pyroptosis. At present, it has been found that these two proteins are involved in inhibiting tumour cell growth and promoting tumour cell death in a variety of system tumours. In addition, some studies also suggest that pyroptosis can promote tumour growth in different kinds of tumour cells [56–58]. This information shows that pyroptosis has dual roles of promoting and inhibiting tumours, and it is necessary to further study the relationship between pyroptosis and tumorigenesis and progression. The occurrence and development of HNSCC are inseparable from inflammation. NOD-like receptor 3 (NLRP3) inflammatory bodies are mainly composed of the receptor protein NLRP3, junction protein ASC, and effector protein caspase-1, which represents the most characteristic inflammatory body [59, 60]. It has been found that the expression of NLRP3 inflammatory bodies is upregulated in HNSCC. At the same time, blocking NLRP3 inflammatory bodies can reduce the number of tumour stem cells (CSCs) in HNSCC cell lines and in the TGFBR1/Pten2cKO mouse HNSCC model. Therefore, NLRP3 inflammatory bodies can be used as a potential target for CSCs in HNSCC therapy [61]. High expression of NLRP3 is also associated with the growth, invasion, and metastasis of HNSCC, with poor clinical prognosis in patients with oral squamous cell carcinoma (OSCC) treated with 5-FU, and NLRP3 gene knockout increases pyroptosis of 5-FU-induced OSCC cells [61, 62]. Gasdermin agonists may improve the efficacy of cancer immunotherapy. Gasdermin is directly activated by Phe-BF3-mediated desalinization, which provides a robust system for understanding the antitumour immunity stimulated by immunogenic cell pyroptosis [63]. Caspase-1 is upregulated in HNSCC tissues and may be a protumour gene in HNSCC tumour tissues. GSDME expression in HNSCC tumour tissue is upregulated and may be a biomarker for poor prognosis [64–66]. IL-6 is a proinflammatory cytokine secreted by cancer cells that is involved in regulating the proliferation and differentiation of cancer cells. The expression level in HNSCC tumour tissue is low, and inhibition of the IL-6 downstream signalling pathway may enhance the therapeutic effect on tumours with elevated IL-6 levels [66–68].

### Ferroptosis

Ferroptosis is a regulated form of cell death characterized by intracellular iron accumulation, increased reactive oxygen species (ROS), decreased reduced glutathione (GSH), and lipid peroxidation, resulting in an imbalance between oxidation and antioxidation systems. Presently, ferroptosis mainly involves three key mechanisms, including iron accumulation, lipid peroxidation, and the destruction of the antioxidant system [12, 19, 69]. When ferroptosis occurs, a large amount of free Fe2 + accumulates in the cells. Free Fe2 + is highly oxidizing and easily undergoes the Fenton reaction with H2O2, which produces hydroxyl radicals that can cause oxidative damage to DNA, protein, and membrane lipids and promote lipid peroxidation to the damaged cell membrane, leading to cell death [19, 70, 71]. Glutathione peroxidase 4 (GPX4) is the most critical anti-lipid peroxidase and the core regulator of ferroptosis. System Xc- is a heterodimer composed of solute carrier family 7 member 11 (SLC7A11) and solute carrier family 3 member 2 (SLC3A2) on the cell membrane, which allows the passage of cystine and glutamate into and out of the cell. System Xc- transports extracellular cystine into cells, which is then converted into cysteine and becomes the raw material for synthesizing GSH [12, 19]. Because GSH is a necessary cofactor for the GPX4 enzyme to decompose peroxides, System Xc- affects the sensitivity of ferroptosis by affecting intracellular cystine metabolism and the GSH/GPX4 pathway, and inhibiting the expression of System Xc-, especially SLC7A11, can enhance the sensitivity of cells to ferroptosis. Therefore, inhibiting GPX4 and consuming GSH in cells is a potential anticancer strategy [71–73].

Acyl coenzyme A (CoA) synthase long-chain family member 4 (ACSL4) and lysophosphatidylcholine acyltransferase 3 (LPCAT3) regulate polyunsaturated fatty acid (PUFA) synthesis [74, 75]. ACSL4 catalyses the ligation of free PUFAs with CoA to form PUFA-CoAs, which are then esterified to form PUFA-PL. Acetyl-CoA carboxylation to malonyl-CoA requires the catalysis of acetyl-CoA carboxylase (ACC). Therefore, the inactivation of ACSL4, LPCAT3, or ACC inhibits the progression of tumour cell ferroptosis [76–78]. Recent studies have shown that in HNSCC, the expression levels of the ACSL1 and TFRC genes are closely related to the occurrence of tumour cell ferroptosis and the prognosis of patients and are potential targets for the occurrence, development and treatment of HNSCC [79]. The high expression of acyl-CoA synthetase long-chain family member 1 (ACSL1) inhibits the progression of thyroid cancer cells and activates fatty acid metabolism reprogramming during cancer cell metastasis, thereby achieving an antagonizing effect of ferroptosis on tumour cells [79–81]. TFRCs are key transporters of intracellular iron and play a key role in tumour cell ferroptosis through their regulation by the NF2-YAP signalling axis. High expression of TFRC is associated with poor prognosis in patients with HNSCC [79, 82]. Compared with the levels in normal tissues, the ferroptosis driver SOCS1 and inhibitor FTH1 in HNSCC were positively correlated and upregulated. In other words, ferroptosis of HNSCC can be induced by increasing the expression of SOCS1 or decreasing the expression of FTH1 [83].

### Autophagy

Autophagy is an intracellular lysosomal pathway that plays a crucial role in maintaining the dynamic balance of various physiological processes. Autophagy involves the sequential formation of three unique membrane structures: phagosomes, autophagosomes, and autolysosomes. More than 40 autophagy-related genes/proteins (Atgs) play a vital role in the dynamics and process of the autophagy membrane [84–87]. Under stress conditions, phagocytic vesicles or isolation membranes can be formed around damaged organelles, abnormally folded proteins, viruses, and bacteria in the cytoplasm, which are isolated into autophagosomes with bilayer membrane structures, and autophagy is induced by AMP-activated protein kinase (AMPK) and mTOR complex 1 (mTORC1) acting on the mechanistic target of rapamycin kinase (mTOR) [85, 88]. The phosphorylation of the UNC-51-like kinase 1 (ULK1) complex (including ULK1, ATG13, FIP200 and ATG101) interacts with the phosphatidylinositol 3-phosphate kinase (PI3K) complex on autophagosomes. Light chain 3 (LC3) modification forms LC3-II and the ATG5-ATG12- ATG16L complex to promote the formation and maturation of autophagic vesicles. After the formation of the autophagosome, it is transported to the vicinity of the lysosome under the action of lysosomal-associated membrane protein (LAMP), Rab7, and VAMP7 [85, 89]. The outer membrane of an autophagosome fuses with a lysosome to form an autophagy‒lysosome, and its contents are degraded by lysosome-related hydrolase. The degradation products of autophagy‒lysosome can be recycled in the cell to meet the cells’ metabolic needs [90–92].

Autophagy can play a major role in cell survival under adverse conditions, which means that autophagy can promote the growth of tumour cells. Autophagy supports tumorigenesis by maintaining mitochondrial metabolic function and energy levels, and may be necessary for tumour cell growth by inhibiting oxidative damage and maintaining metabolic homeostasis [93, 94]. The purpose of inhibiting the growth of tumour cells can be achieved by inducing tumour cells to activate autophagy. Hypoxia upregulated the expression of circCDR1as and induced autophagy in OSCC. Studies have shown that circCDR1as, as an oncogene, encapsulates miR-671-5p by sponging, inhibits mTOR activity, upregulates the AKT and ERK signalling pathways, and induces autophagy in OSCC cells [95, 96]. Erythroblast macrophage protein (MAEA) is a new type of E3 ubiquitin ligase that regulates autophagy through the AMPK and PI3K/AKT pathways. In HNSCC, TBC1D14 inhibits autophagy by downregulating the expression of MAEA. Overexpression of TBC1D14 inhibits autophagy by reducing the level of Lc3-II protein, increasing the level of p62 protein, and inhibiting the formation of autolysosomes, thus playing a role in anti-HNSCC metastasis [97–99]. Circ-PKD2 can increase the sensitivity of oral squamous cell carcinoma to cisplatin by inhibiting miR-646. Circ-PKD2 increases the level of Atg13, inhibits tumour proliferation and activates autophagy in OSCC cells by promoting the formation of autophagosomes and the activation of LC3II/I and other proteins [100, 101].

### Cuproptosis

Copper ions are an indispensable cofactor for all organisms, but when their concentration exceeds a certain threshold, they will have a toxic effect on the body [102]. Tsvetkov P et al. discovered a new method of cell death that is dependent on copper ions and is regulated in the cell body, termed cuproptosis; copper ions directly bind to the fatty acylation components in the tricarboxylic acid cycle, resulting in abnormal accumulation of rich acylated proteins and loss of iron-sulfur cluster proteins, resulting in a protein toxic stress reaction and eventually leading to cell death [103]. This is different from the death mechanism that we know. Heavy metal ions are essential micronutrients for the human body, but insufficient or excessive metal abundance will lead to cell death [104]. At present, we have identified seven critical genes in the regulatory mechanism of cuproptosis, namely, ferredoxin 1 (FDX1), lipoic acid synthase (LIAS), lipoyl transferase-1 (LIPT1), dihydrolipoamide dehydrogenase (DLD), dihydrolipoamide-acetyltransferase (DLAT), pyruvate dehydrogenase E1 alpha (PDHA1) and pyruvate dehydrogenase β subunit gene (PDHB), and three harmful regulatory genes, namely, metal-regulated transcription factor (MTF), glutaminase (GLS) and cyclin-dependent kinase inhibitor 2 A (CDKN2A) [105]. Mitochondrial lipoylation is a posttranslational modification of lysine, which allows entry into the tricarboxylic acid (TCA) cycle through regulation by dihydrothiooctylamide branched chain transferase E2 (DBT), glycine lytic system protein H (GCSH), dihydrothiooctylamine-succinyltransferase (DLST) and DLAT. Copper directly binds to DLAT to promote the disulfide bond-dependent aggregation of lipid-based DLAT [106, 107].

In the Wilson disease mouse model, the loss of the copper exporter ATPase copper transporting beta (ATP7B) leads to excessive accumulation of copper and copper damage in the ageing liver, suggesting that drug inhibition of mitochondrial respiration may be a strategy against the disease [103]. Some experiments have shown that compared with normal oral tissues, the tissues of HNSCC patients express high levels of ATP7B, ATP7B in mitochondria and excrete copper from the cytoplasm to the extracellular space [108, 109]. In the differential expression analysis of oral squamous cell carcinoma and adjacent tissues, LIAS and PDHB were inhibited in cancer tissues, while GLS and CDKN2A were promoted in cancer tissues. This shows that the cuproptosis of tumour cells is inhibited, and the growth of tumour cells is promoted in OSCC [110]. Intratumor copper can regulate the expression of PD-L1, affect the immune escape of tumours, and then affect the efficacy of immunotherapy [111]. Mutations in the mitochondrial ribosomal gene MRPS7 affect mitochondrial respiratory chain dysfunction, and high expression of MRPS7 is associated with poor prognosis in patients with HNSCC [112, 113]. Studies have shown that high-dose Cu2 + exposure induces oxidative stress by increasing the levels of ROS and protein carbonyl compounds and reducing the GSH content and mRNA and enzyme activities, resulting in mitochondrial membrane potential depolarization, Cyto C release, cleavage of caspase-9 and caspase-3, increased BAK and BAX levels, decreased BCL2 levels and induction of tumour cell apoptosis [114]. The effect of elesclomol (ES) on cancer cells is to increase the level of Cu2 + in mitochondria, reduce the expression of ATP7A, lead to Cu2 + retention and the accumulation of ROS, promote SLC7A11 degradation, further enhance oxidative stress in cells and thereby induce tumour cell cuproptosis [115].

## Modulation of cell death in HNSCC

**Fig. 2:**
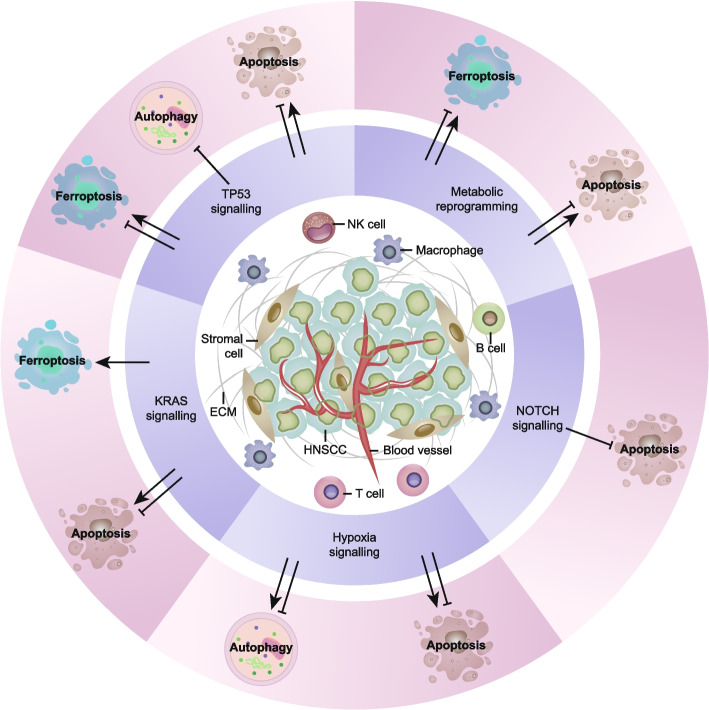
Modulation of cell death pathways in HNSCC. The tumour microenvironment of HNSCC is a complex ecosystem composed of cellular components (such as malignant cells, immune cells and stromal cells), extracellular matrix and interstitial fluid (such as blood vessels, cytokines, chemokines and growth factors). The interaction between cancer cells and invasive immune cells determines the progression and therapeutic effect of the tumour. ECM, extracellular matrix; NK, natural killer; TP53, tumour protein p53

During the growth of HNSCC cells, a complex signal communication network has emerged to promote a collaborative environment that supports tumour proliferation and spread [116]. The TME is a complex ecosystem composed of cellular components (such as malignant cells, immune cells, and interstitial cells) and noncellular components (such as extracellular matrix, blood vessels, cytokines, chemokines, and growth factors) [117]. Various oncogenes or tumour suppressor signals in the TME determine the sensitivity of cell death patterns in HNSCC (Fig. 2). In this section, we focus on some of the most common HNSCC functions that affect cell death signals.

### TP53 signalling

The inactivation of tumour suppressor genes and the activation of oncogenes are key to the occurrence and development of HNSCC [118]. To date, the TP53 gene is the most commonly mutated gene in human cancer [119]. The mutation rate of the TP53 gene in human tumour cells has increased significantly from 10% (haematopoietic stem cell malignant tumour) to 50–70% (ovarian, colorectal, and head and neck cancer) [120]. Once the TP53 gene is mutated, the tumour suppression function of the wild-type TP53 gene will be lost, and many individual oncogenes will be active [121]. The TP53 gene encodes a tumour suppressor protein with transcriptional activation, DNA binding, and oligomerization domains. It is a tumour suppressor gene and can induce cell cycle arrest, apoptosis, and senescence. Mutation of the TP53 gene is related to many kinds of human cancers [122]. Cancer can be detected earlier and more accurately by extracting DNA from plasma to detect TP53 mutations [123]. Some studies have shown that high expression of TP53 plays a promoting role in the initial carcinogenesis of oral cancer [124].

**Fig. 3:**
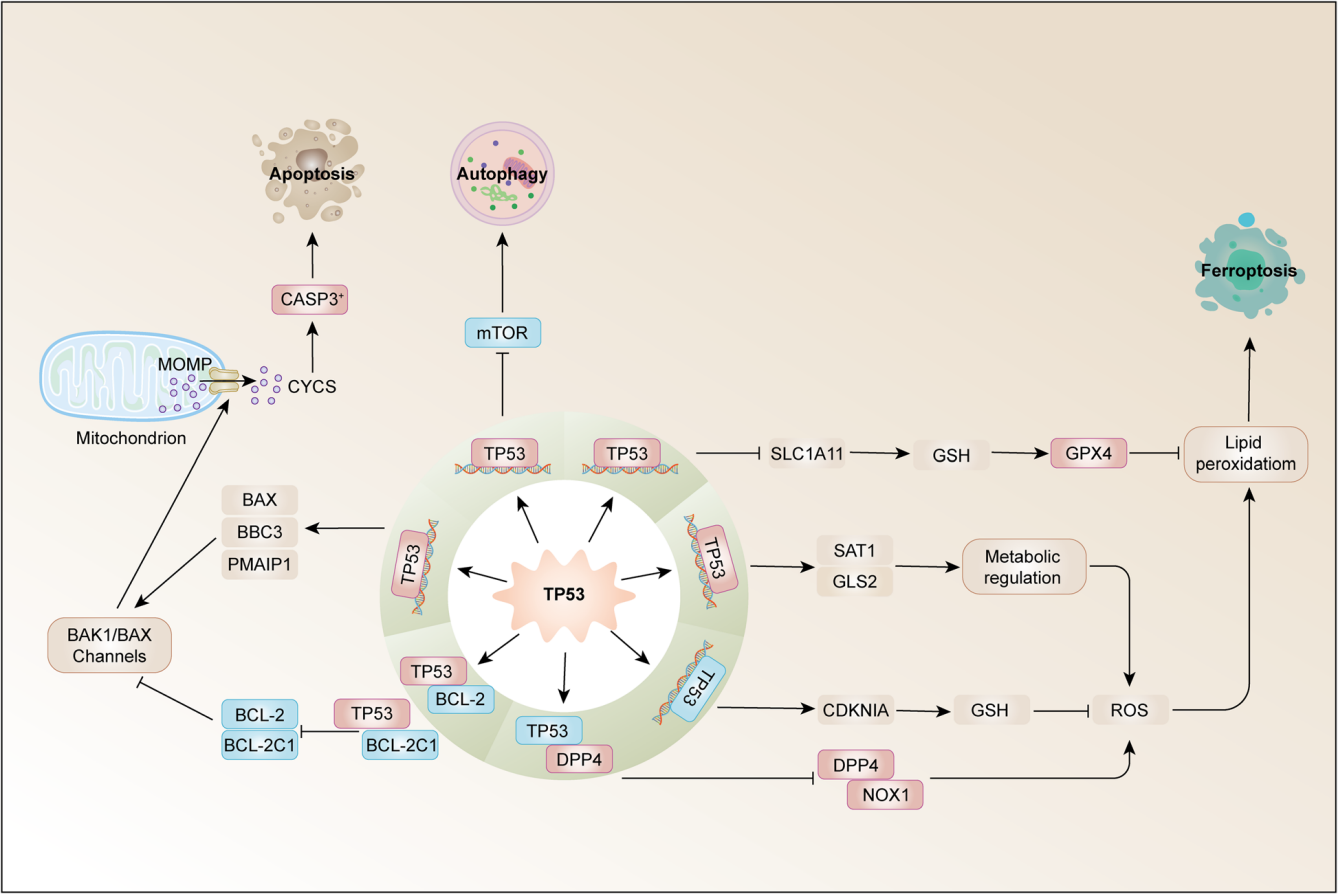
TP53 in HNSCC cell death. Tumour protein p53 (TP53) acts on mitochondria. MOMP inhibits BCL-2 and BCL-2L1, as antiapoptotic members, and activates BAX, BBC3 (also known as PUMA) and PMAIP1 (also known as NOXA), which are proapoptotic members of the Bcl2 family, thus triggering TP53-dependent apoptosis. P53 can also downregulate the mTOR signalling pathway to promote autophagy. TP53 induces iron death by inhibiting the expression of SLC7A11 or directly acting on diamine acetyltransferase SAT1 and mitochondrial glutaminase GLS2. TP53 inhibits iron death by inhibiting the activities of dipeptidyl peptidase 4 (DPP4) and NADPH oxidase 1 (NOX1) or inducing the expression of cyclin-dependent kinase inhibitor 1 A (CDKN1A). BAK1, BCL-2 homologous antagonist/killer 1; BBC3, BCL-2-binding component 3; CDKN1A, cyclin-dependent kinase inhibitor 1 A; CYCS, cytochrome C, somatic; GLS2, glutaminase 2; GPX4, glutathione peroxidase 4; GSH, glutathione; mTOR, mechanistic target of rapamycin kinase; PMAIP1, phorbol-12–myristate-13 acetate-induced protein 1; ROS, reactive oxygen species; SLC7A11, solute carrier family 7 member 11; SAT1, spermidine/spermine N1-acetyltransferase 1

TP53 mutation occurs in the early stage of cancer. HPV-negative HNSCC has a typical TP53 mutation, while in HPV-positive cases, TP53 is downregulated by HPV oncogene E6 and usually does not mutate [125, 126]. During DNA damage and oncogene activation (carcinogenic stress), TP53 transcription factor, as a cell stress receptor, is activated, along with cyclin-dependent kinase inhibitor 1 A (CDKN1A, coding p21), which triggers cell cycle arrest and senescence, as well as BCL2-related X, apoptosis regulatory factor (Bax), TP53-upregulated apoptosis regulator (PUMA; also known as BBC3) and NOXA (also known as PMAIP1), thus triggering apoptosis [127–129] (Fig. 3). In malignant lesions, part of the TP53 protein is located in mitochondria. MOMP inhibits the antiapoptotic members Bcl2, Bcl-xl, and Mcl-1 and activates the proapoptotic Bak and Bax proteins of the Bcl2 family, thus triggering TP53-dependent apoptosis [130, 131]. P53 can also downregulate the mTOR signalling pathway to promote autophagy and induce the expression of genes, such as DRAM, PUMA, BAX, ISG20L1, and EI24, to promote autophagy [132, 133].

TP53 causes apoptosis, and the activation of TP53 plays a vital role in inducing ferroptosis in some tumour cells [134]. The activation of TP53 can effectively reduce the expression of SLC7A11 at the mRNA and protein levels, while knockout of the p53 gene can relieve the inhibition of SLC7A11. [135] Inhibiting the expression of SLC7A11 by activating p53 can reduce the activity of System Xc- and eventually lead to ferroptosis [136]. In addition to inhibiting the movement of the System Xc- system, TP53 can directly act on diamine acetyltransferase SAT1 and mitochondrial glutaminase GLS2, regulate the metabolic process of glutamine and indirectly induce ferroptosis [137, 138]. Recent studies have found that erastin can activate TP53 and promote ferroptosis in cells. In the process of ferroptosis caused by erastin, the intracellular ROS produced can initiate TP53, resulting in a significant increase in the level of TP53 transcripts, thus further increasing the level of intracellular ROS [139]. However, in some cases, TP53 can also reduce the sensitivity of cells to ferroptosis. TP53 can activate p21 and delay ferroptosis in a transcription-dependent manner [140]. TP53 can also inhibit ferroptosis by inhibiting the activities of dipeptidyl peptidase 4 (DPP4) and NADPH oxidase 1 (NOX1) or inducing the expression of cyclin-dependent kinase inhibitor 1 A/p21 (CDKN1A/p21) [140, 141]. TP53 is at the core of the ferroptosis signalling network. On the one hand, it can be activated by erastin to enhance the sensitivity of cells to ferroptosis and inhibit tumorigenesis. On the other hand, TP53 protects normal cells from various stress factors. When some metabolic stress occurs, TP53 can reduce the sensitivity of cells to ferroptosis and maintain normal physiological function. Generally, the role of TP53 in the ferroptosis signalling network is very complex, and its specific mechanism in cancer treatment needs to be further studied [142].

### KRAS signalling

Kirsten rat sarcoma virus oncogene (KRAS) mutation is one of many tumours’ most common carcinogenic factors. The KRAS gene encodes the KRAS protein. KRAS is a small GTP enzyme that acts as a molecular switch for various cellular processes by coupling membrane growth factor receptors with intracellular signalling pathways and transcription factors [143]. When KRAS is mutated, it loses the GTP hydrolase activity and then continues to be activated, promoting cell proliferation and carcinogenesis. We have been looking for drugs that can interfere with the binding of KRAS to GTP to block the carcinogenic effect of the mutant KRAS gene. Nevertheless, because of the particularity of the KRAS protein structure, there is no effective drug to treat KRAS mutant tumours thus far. Epidermal growth factor receptor (EGFR) is the upstream signalling pathway of KRAS [144, 145]. Through the study of targeted EGFR inhibitors, the upstream signalling pathway can be blocked to block the mutant expression of KRAS. EGFR is overexpressed in 80–90% of HNSCC and is associated with poor prognosis and treatment outcomes. Activation of KRAS signalling can promote metabolic reprogramming and autophagy in tumour cells to maintain proliferation and inhibit apoptosis [146–148]. Cancer cells with mutant RAS (including KRAS), such as HNSCC cells, are sensitive to the induction of ferroptosis, which may be due to the mutated expression of iron metabolism genes mediated by RAS, such as transferrin receptor TFRC, FTH1 and FTL. In HNSCC patients with overexpression of EGFR and cyclin D1, the combined effect of afatinib and palbociclib induces ageing by reducing the ATP pool, reducing antioxidant enzymes, and increasing the production of ROS, thus causing metabolic changes and enhancing the combined antitumour effect [149]. EGFR inhibitors are the only approved targeted drugs, but their efficacy is limited, and intrinsic and acquired drug resistance mechanisms remain unresolved [150].

### NOTCH signalling

In HNSCC, NOTCH signalling pathway genes are changed at the transcriptional level, and NOTCH1 has been identified as a tumour suppressor gene [151, 152]. The NOTCH pathway transmits signals through transcriptional activation of the target genes HES1 and HEY1. The prognosis of patients with NOTCH1 mutation is poor, in which the direct downstream targets HES1 and HEY1 are overexpressed [153, 154]. HES, the target gene of the classical Notch signalling pathway, inhibits the expression of PTEN. When the NOTCH signal is suppressed, it downregulates the expression of HES, and HES suppresses PTEN and upregulates the expression of PTEN, which inhibits PI3K-AKT-mediated survival signal transduction and leads to tumour cell apoptosis [155, 156]. The activity of Notch is related to the inhibition of HPV E6 and E7 protein expression, which may provide additional selection pressure for the loss of NOTCH in HPV + HNSCC [157, 158]. In mature epithelium, the expression of p63 is the highest in basal epithelial cells, where it acts as an inhibitor of NOTCH1 expression and is downregulated during terminal differentiation, consistent with the upregulation of NOTCH1 [159]. ADAM10 and AJUBA inhibit HNSCC by promoting NOTCH receptor signal transduction in a haploid-deficient manner [160]. CD44 + cells overexpress HIF-1α in HNSCC. Inhibiting the expression of HIF-1α can inhibit the expression of NOTCH1 and block the metastasis rate and chemotherapy resistance of HNSCC cells. Therefore, NOTCH1 plays an essential role in maintaining the characteristics of HNSCC cells, promoting the malignancy of HNSCC cells and drug resistance to chemotherapy and radiation [161].

### Hypoxia signalling

Hypoxia can aggravate tumour formation and treatment resistance. In HNSCC, hypoxia may lead to adverse reactions to radiotherapy and poor prognosis [162]. In solid tumours, tumour cells maintain their adaptation to hypoxia mainly by regulating hypoxia-inducible factor (HIF). The HIF family consists of three members, namely, HIF-1, HIF-2, and HIF-3, which can sense the changes in oxygen concentration in the microenvironment and regulate the pathway for cells to adapt to hypoxia [163, 164]. HIF-1 is a heterodimeric protein composed of the HIF-1α and HIF-1β subunits. Under normoxic conditions, HIF-1α is easily degraded by the ubiquitin protease system. At the same time, the degradation of HIF-1α is inhibited during hypoxia, which leads to its continuous accumulation, which makes cancer cells adapt to a hypoxic environment and provides conditions for the proliferation, invasion, and metastasis of cancer cells [164]. HIF-1 also interacts with P53 to promote caspase-3-dependent apoptosis [165, 166].

Hypoxia has a direct effect on a variety of cell death mechanisms and regulates the interaction between different pathways. During hypoxic stress, Bcl-2/adenovirus E1B 19-kDa protein interacting protein 3 (BNIP3) may induce autophagy by isolating Bcl2 or xl from the Bcl2 or Xl-Beclin1 complex, and then the shifted Beclin-1 can trigger autophagy [167]. In addition, caspase-mediated Beclin-1 cleavage inhibits autophagy and enhances apoptosis by promoting the mitochondrial release of proapoptotic factors and enhancing caspase-9 activity, a joint event in HNSCC [168, 169].

### Metabolic reprogramming

Considering the massive demand for energy and biomass for cell survival, cancer cells show unique metabolic characteristics compared with normal cells. By participating in many core metabolic pathways, including glycolysis, mitochondrial metabolism, and the anabolism/catabolism of fats and amino acids, external signals are transmitted into cells to support cell survival [170, 171]. In other words, a series of changes cause tumour cells to reregulate adenine nucleoside triphosphate (ATP) production or increase glucose uptake to promote energy production. Under sufficient oxygen, tumour cells promote the rapid energy supply of glycolysis due to the change in the microenvironment, which is called the “Warburg effect” [172, 173]. Metabolic reprogramming in the TME was confirmed by analysing the metabolic-related gene prognostic index (MRGPI) in HNSCC.

System Xc- reverses glutamate transport and transduces cystine into cells. GPX4 catalyses the sulfhydryl binding of lipid peroxides (L-OHO) to reduce glutathione and converts harmful substances into nontoxic fatty alcohols (L-OH), thus preventing the ROS chain reaction and avoiding ferroptosis [174–176]. The upregulation of System Xc- expression may be related to chemotherapy resistance and tumour growth [177]. The absence of GPX4 increases phospholipid hydrogen peroxide and promotes lipoxygenase-mediated lipid peroxidation and eventually leads to ferroptosis [178]. Ferroptosis inhibitor protein 1 (FSP1) is an oxidoreductase that reduces coenzyme Q10 and produces a lipophilic free radical capture antioxidant to prevent lipid peroxidation and ferroptosis [179]. The tumour suppressor BAP1 enhances ferroptosis by regulating the expression of cystine transporter SLC7A11, thus improving the control of tumour growth [180, 181]. The concentration of glutathione (GSH) measured in metastatic tumours was higher than that of the corresponding primary tumours, indicating that GSH metabolism may affect the formation of HNSCC metastasis. In HPV-negative HNSCC, nonfunctional p53 leads to a decrease in mitochondrial OXPHOS and the prioritizing of glycolysis. Therefore, HPV-positive HNSCC may be more sensitive to mitochondrially targeted therapies, such as mitogens. Thus, the HPV oncoprotein E7 enhances ceramide-mediated mitosis and lethal mitosis to cope with chemotherapy-induced mitochondrial damage [182–184].

## Therapy options in HNSCC

**Fig. 4:**
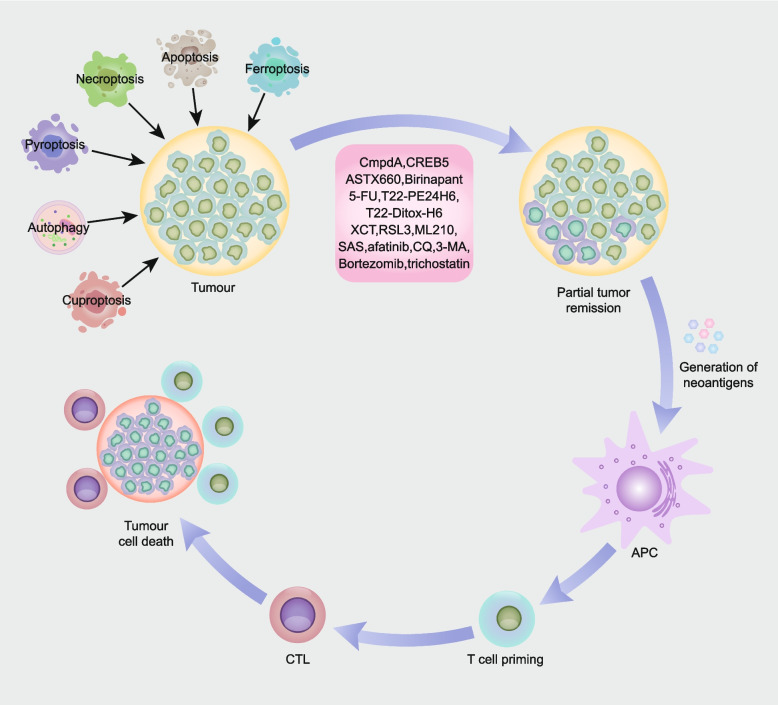
Induction of cell death by therapeutic regimens evokes antitumour immune responses. Therapeutic modalities, including apoptosis, necroptosis, pyroptosis, ferroptosis, autophagy, and cuproptosis, induce death in cancer cells. Apoptosis, necroptosis, pyroptosis, ferroptosis, autophagy and cuproptosis in cancer generate abundant neoantigens, which are processed by antigen-presenting cells to promote the formation of antigen-specific cytotoxic T lymphocytes (CTLs), thereby evoking antitumour immunity. Agents that target apoptosis, necroptosis, pyroptosis, ferroptosis, and autophagy are noted in the figure (apoptosis inhibitor: CmpdA, CREB5; necroptosis inducer: ASTX660, birinapant; pyroptosis inducer: 5-FU, T22-PE24H6, T22-Ditox-H6; ferroptosis inducer: RSL3, ML210, SAS; ferroptosis inhibitor: XCT; autophagy inhibitor: afatinib, CQ, 3-MA, bortezomib, trichostatin)

Surgical resection, radiotherapy, and chemotherapy are the main treatments for HNSCC, and identifying the most effective treatment and optimizing function preservation are our ideal goals. Immunogenic cell death (ICD) is a process whereby tumour cell death is induced and activates the body’s immune system to fight against dead cell antigens and endow dead tumour cells with immunogenicity [185, 186]. When ICD occurs, dead cells produce new antigenic epitopes and release DAMPs. Antigen-presenting cells (APCs) recognize and phagocytize dead cell antigens and present them to T cells, promoting the formation of antigen-specific cytotoxic T lymphocytes (CTLs) and activating the adaptive immune response to recognize and clear tumour antigens, resulting in long-term antitumour immune effects [187, 188] (Fig. 4).

### Apoptosis in targeted therapy

The transcription factor NF-κB can regulate apoptosis and chemotherapy resistance [189]. NF-κB signal transduction can promote cisplatin resistance and increase the sensitivity of cancer cells to cisplatin therapy. Active NF-κB signalling in HNSCC cells can promote histone deacetylation and heterochromatin production to induce drug resistance to chemotherapy. Furthermore, targeted inhibition of histone deacetylase to destroy tumour drug resistance caused by NF-κB may be a therapeutic strategy [190, 191]. Other studies have shown that NF-κB phosphorylation at the serine 536 position plays a crucial role in regulating cisplatin resistance [192]. IKK phosphorylated NF-κB at serine 536, and NF-κB p65 gene knockout or overexpression of the NF-κB p65-serine 536 A mutant made drug-resistant cells sensitive to cisplatin. Therefore, blocking the activity of IKK/NF-κB with the IKK β inhibitor CmpdA may also be a strategy to improve the efficacy of cisplatin in HNCSS [193]. When APAF1 gene knockout or CRISPR-mediated caspase-9 deletion occurs, mitochondrial-dependent caspase activity is blocked. MOMP induces the activation of NF-κB and the production of TNF by degrading the IAP protein and upregulating the expression of NF-κB-induced kinase. Moreover, MOMP combined with caspase inhibition can transfer mitochondrial DNA to the cytoplasm through Bak1-Bax macropores, activate signalling in the cytoplasm, and promote the secretion of type I interferon, thus triggering the innate immune response [194–196]. CREB5 increases mitochondrial activity and ATP production through TOP1MT, promotes the expression of the antiapoptotic protein Bcl2, and increases the Bcl2/Bax ratio, thus inhibiting the mitochondrial apoptotic pathway and promoting cisplatin resistance in HNSCC cells. Dual targeting of CREB5 and TOP1MT is also a promising strategy to combat cisplatin resistance [197].

### Necroptosis in targeted therapy

Approximately half of the necrosis in HNSCC can be attributed to necroptosis [36]. TNFα, TRAIL, or other death agonists bind to their receptors, activate RIPK1 and MLK1 and induce necroptosis, resulting in cell death [198]. RIP3 is a crucial regulator of necroptosis caused by TNF. Loss of RIP3 expression may reduce the sensitivity of cells to necroptosis stimulation, which in turn promotes cell survival and tumorigenesis [199]. Fumaric acid is a tumour metabolite. In many cancers, fumaric acid accumulation is caused by fumarate hydratase (FH) mutations [200]. The expression of EB virus (LMP1) in HNSCC leads to a decrease in fumaric acid and α-KG and inhibits the expression of RIP3 by DNA methylation, thus inhibiting necroptosis. This approach provides a new choice for the diagnosis and treatment of HNSCC [39]. CIAP/XIAP can inhibit cell death mediated by the mitochondrial and caspase-3 pathways, while the IAP1/XIAP antagonist ASTX660 can increase the sensitivity of HNSCC cells to TNF-α and TRAIL by promoting the expression of TP53 protein and induce necrotic apoptosis of HPV (-) cells by the cleavage of caspase-3 and caspase-8 [201, 202]. At the same time, IAP1/XIAP antagonists combined with radiotherapy had a more significant effect. This finding suggests that IAP1/XIAP antagonists, especially in combination with radiotherapy, are a potential treatment for HNSCC [203]. Studies have shown that FADD amplification is more common in HNSCC than in other cancers, with upregulation of FADD in HNSCC cell lines and minimal expression of BIRC3. CIAP can inhibit TNFα-FADD signalling to promote necroptosis [204]. The Smac analogue birinapant combined with TNFα promoted the degradation of CIAP1, the product of BIRC2, and activated caspase-8-mediated apoptosis and MLKL-mediated necroptosis in HNSCC. At the same time, birinapant plus radiotherapy may aggravate the death of HNSCC, which is a new direction in the treatment strategy [204].

### Pyroptosis in targeted therapy

Among the pyroptosis pathways, the executive protein GSDME may be associated with chemoresistance in HNSCC [205, 206]. As a tumour suppressor, GSDME enhances antitumour immunity by activating cellular pyroptosis. In mice, the upregulation of GSDME can enhance the phagocytosis of macrophages and increase the number and function of tumour-infiltrating natural killer cells and CD8 + T lymphocytes [207]. In OSCC, tumours with high expression of GSDME increased the number of infiltrating CD8 + T cells, granzyme B, and M1 phenotypic macrophages. At the same time, cellular pyroptosis induced by GSDME-mediated chemotherapy played an essential role in the antitumour response [206, 208]. The activation of pyroptosis can trigger the efficacy of various antineoplastic drugs. Paclitaxel and cisplatin can activate GSDME and release inflammatory bodies, transforming the cell death pathway from apoptosis to pyroptosis, thus inhibiting the proliferation of cancer cells [209–211]. 5-Fluorouracil (5-FU) converts caspase-3-dependent apoptosis induced by chemotherapy drugs to pyroptosis by triggering the caspase-3/GSDME signalling pathway [212]. In the HNSCC mouse model with high expression of CXCR4, T22-PE24H6 and T22-Ditox-H6 nanotoxins showed strong antitumour effects by inducing caspase-3/GSDME-dependent pyroptosis. At the same time, recurrent or metastatic HNSCC may be sensitive to nanotoxin therapy. This mechanism of cell death, which triggers alternative apoptosis, is also a new therapeutic strategy for chemotherapy and radiotherapy resistance in patients with HNSCC [213–216].

### Ferroptosis in targeted therapy

Ferroptosis inducers can act with more traditional drugs, such as cisplatin, to inhibit tumour growth in mouse models of head and neck cancer [217]. SLC7A11 (XCT) is a Na+-dependent cysteine/glutamate exchanger encoded by the SLC7A11 and SLC3A2 genes that can promote the synthesis of glutathione [218]. Inhibiting the activity of XCT in drug-resistant HNSCC cells induces glutathione depletion, thus promoting ferroptosis and enhancing the cytotoxicity of cisplatin in drug-resistant HNSCC cells [217, 219]. IFNγ released by CD8 + T cells induces ferroptosis of tumour cells by activating the JAK-STAT1 pathway, inhibiting cysteine uptake, reducing LOOH to nontoxic LOH, and downregulating the expression of SLC3A2 and SLC7A11 [220–222]. For example, GPX4 inhibitory molecules such as RSL3 and ML210 help to inhibit enzyme activity, resulting in excessive lipid peroxidation, leading to ferroptosis in tumour cells. [223] System Xc- inhibitors such as sulfasalazine and erastin can block the activity of System Xc- and entry of cystine into cells, thus inducing ferroptosis in tumour cells [19]. HMGB1 is related to the immunogenicity of tumour cell ferroptosis. Tumour cells that produce ferroptosis acetylate HMGB1 and release HMGB1 through autophagy. At the same time, a large amount of HMGB1 was found in the serum and tissues of HNSCC, indicating that HMGB1 stimulates the chemotaxis of regulatory T cells (Tregs) and promotes their immunosuppressive function [224, 225]. Ferroptosis can also induce the expression of cyclooxygenase-2 and the release of prostaglandin E2 (PGE2). In addition, the release of PGE2 is positively correlated with the immune escape of tumour cells [226].

### Autophagy in targeted therapy

In HNSCC, IFNα has the function of immune regulation and inhibition of tumour cell proliferation [227]. On the one hand, IFNα can resist expansion and induce apoptosis and can also activate autophagy in human nasopharyngeal carcinoma cells. This autophagy has a cytoprotective effect and can promote IFNα-mediated apoptosis. On the other hand, IFNα combined with the autophagy inhibitors wortmannin and HCQ can increase the antitumour activity of IFNα, thus inhibiting autophagy, which is also a new strategy for the treatment of nasopharyngeal carcinoma [228, 229]. Afatinib is a second-generation tyrosine kinase inhibitor (TKI) that exerts its antitumour effect by inhibiting mTORC1-mediated autophagy in HNSCC [230]. Afatinib induced upregulation of Redd1 expression through ROS stimulation, resulting in downregulation of TSC1 and low conversion of LC3B-II, and then it inhibited mTORC1 to promote autophagy in HNSCC. [231] The autophagy inhibitors CQ and 3-MA block autophagy, significantly enhancing the sensitivity of tumour cells to afatinib-induced apoptosis. At the same time, the combination of afatinib and autophagy inhibitors may eliminate HNSCC tumour stem cells, which is also a potential clinical treatment [231, 232]. AMPK promotes autophagy by activating ULK1, while mTOR inhibits autophagy by phosphorylating ULK1 and disrupting the interaction between ULK1 and AMPK [233]. Inhibition of histone deacetylase 6 (HDAC6) activity can induce autophagy by affecting the activity of the autophagy promoter ULK1 in HNSCC cells through mTOR. Inhibition of HDAC6 can enhance the cytotoxicity of the proteasome inhibitor bortezomib to induce apoptosis. At the same time, the combination of bortezomib and the histone deacetylase inhibitor trichostatin inhibited HDAC6 activity, reduced autophagy induction, and significantly enhanced bortezomib-induced apoptosis of HNSCC cells. Therefore, targeting HDAC6 for chemotherapy-resistant HNSCC is a very effective treatment strategy [234, 235].

### Cuproptosis in targeted therapy

It was found that the content of copper in serum and tumour tissues increased in patients with breast cancer, cervical cancer, ovarian cancer, lung cancer, gastric cancer, bladder cancer, thyroid cancer, oral cancer, and pancreatic cancer, which was closely related to the stage and progression of some cancers. [236–239]. The characteristics of copper enrichment in tumour tissues are becoming attractive targets in developing anticancer drugs. Copper chelators such as D-penicillamine, tetrathiomolybdate (TTM), and triamolybdate reduce the bioavailability of copper by directly combining with copper. Copper ion carriers such as clioquinol, 22-carbon hexanoic acid, thiosemicarbazone, and disulfiram (DSF) can increase intracellular copper levels. Through the production of ROS, proteasome inhibition and induction of apoptosis play antitumour roles [240]. Copper induces necroptosis and toxic damage through ROS-dependent DNA damage, and the necroptosis inhibitor Nec-1 or z-VAD-FMK can alleviate Cu2+-induced death [241]. Targeting the regulation of cellular ferroptosis induced by intracellular copper ions can be used to improve the sensitivity of the antitumour effect. Excess copper accumulation leads to cuproptosis by inducing DLAT aggregation and enhancing ROS and inactivating GPX4. Overloaded iron ions induce potent ferroptosis, which synergizes with cuproptosis and DOX-induced apoptosis to kill tumour cells [242]. The combination of DSF and antineoplastic drugs can enhance the antitumour effect and have a far-reaching impact on treating human cancer. When nasopharyngeal carcinoma cell lines and nonmalignant nasopharyngeal epithelial cells were treated with DSF and Cu2+, cell viability was significantly inhibited compared with that of DSF alone, and RNA-seq analysis showed that the ferroptosis pathway was involved in DSF/Cu-induced cell death. Seven ferroptosis-related genes were changed considerably, which confirmed the copper-dependent ferroptosis induction of DSF on nasopharyngeal carcinoma cells [243]. Although there are few studies on targeted therapy for HNSCC cuproptosis, with more in-depth research, there will be a new breakthrough in the treatment of HNSCC.

## Conclusions and perspectives

Since an increasing number of RCDs have been discovered, the mechanism of RCD and its interaction with tumour cells have been gradually revealed. Many HNSCC treatment strategies induce cell death and prevent tumour growth. In this context, targeting apoptotic cell death appears to be an increasingly promising strategy to improve therapeutic outcomes in cancer therapy. However, the induction of tumour cell death is accompanied by the end of normal cells, and apoptosis, necroptosis, pyroptosis, autophagy, ferroptosis, and cuproptosis may play an antitumour role under different conditions and may also promote the growth of tumour cells. Therefore, we must explore a more targeted and personalized treatment to resist tumour cell proliferation. Fully understanding the development of HNSCC and apoptosis, necroptosis, pyroptosis, autophagy, and the mechanism of action of ferroptosis and cuproptosis for the treatment of HNSCC will provide new therapeutic targets and strategies that, combined with radiotherapy, chemotherapy, and targeted therapy, can effectively eliminate tumour cells and protect healthy cells at the same time, which may prolong the survival of HNSCC patients and is also a treatment direction of great research significance.

## Data Availability

Not applicable.
